# Decoding Genetic Features and Antimicrobial Susceptibility of *Pseudomonas aeruginosa* Strains Isolated from Bloodstream Infections

**DOI:** 10.3390/ijms23169208

**Published:** 2022-08-16

**Authors:** Tomasz Bogiel, Dagmara Depka, Mateusz Rzepka, Agnieszka Mikucka

**Affiliations:** Microbiology Department, Ludwik Rydygier Collegium Medicum in Bydgoszcz, Nicolaus Copernicus University in Toruń, 85-094 Bydgoszcz, Poland

**Keywords:** bacteremia, bloodstream infections, BSIs, *Pseudomonas aeruginosa*, *Pseudomonas aeruginosa* virulence, toxins, virulence, virulence genes

## Abstract

*Pseudomonas aeruginosa* is a Gram-negative rod and an etiological factor of opportunistic infections. The infections of this etiology appear mostly among hospitalized patients and are relatively hard to treat due to widespread antimicrobial resistance. Many virulence factors are involved in the pathogenesis of *P. aeruginosa* infection, the coexistence of which have a significant impact on the course of an infection with a particular localization. The aim of this study was to assess the antimicrobial susceptibility profiles and the frequency of genes encoding selected virulence factors in clinical *P. aeruginosa* strains isolated from bloodstream infections (BSIs). The following genes encoding virulence factors of enzymatic activity were assessed: *lasB*, *plC H*, *plC N*, *nan1*, *nan2, aprA* and *phzM*. The frequency of the genes encoding the type III secretion system effector proteins (*exoU* and *exoS*) and the genes encoding pilin structural subunits (*pilA* and *pilB*) were also investigated. The occurrence of virulence-factor genes was assessed using polymerase chain reactions, each in a separate reaction. Seventy-one *P. aeruginosa* strains, isolated from blood samples of patients with confirmed bacteremia hospitalized at the University Hospital No. 1 of Dr. Antoni Jurasz in Bydgoszcz, Poland, were included in the study. All the investigated strains were susceptible to colistin, while the majority of the strains presented resistance to ticarcillin/clavulanate (71.8%), piperacillin (60.6 %), imipenem (57.7%) and piperacillin/tazobactam (52.1%). The presence of the *lasB* and *plC H* genes was noted in all the tested strains, while the *plC N*, *nan2*, *aprA*, *phzM* and *nan1* genes were identified in 68 (95.8%), 66 (93.0%), 63 (88.7%), 55 (77.5%) and 34 (47.9%) isolates, respectively. In 44 (62.0%) and 41 (57.7%) strains, the presence of the *exoU* and *exoS* genes was confirmed, while the *pilA* and *pilB* genes were noted only in 14 (19.7%) and 3 (4.2%) isolates, respectively. This may be due to the diverse roles of these proteins in the development and maintenance of BSIs. Statistically significant correlations were observed between particular gene pairs’ coexistence (e.g., alkaline protease and neuraminidase 2). Altogether, twenty-seven distinctive genotypes were observed among the studied strains, indicating the vast variety of genetic compositions of *P. aeruginosa* strains causing BSIs.

## 1. Introduction

*Pseudomonas aeruginosa* is an opportunistic Gram-negative non-fermenting rod and is a typical bacterial pathogen often found in a hospital environment, which frequently causes intra-ward nosocomial outbreaks. *P. aeruginosa* strains are responsible for a wide range of human infections, occurring mostly amongst hospitalized patients [1,2]. Thus, *P. aeruginosa* rods represent one of the bacteria most frequently isolated from clinical specimens. Their increasing isolation incidence and role in nosocomial infections with different localizations are underlined by an involvement in respiratory, urinary tract and wound infections and bacteremia, where the last is usually a consequence of infections in a primary localization. Bloodstream infections (BSIs) caused by *P. aeruginosa* strains are the most threatening and one of the most important issues [2,3,4] concerning intensive-care and pediatrics-unit patients.

*P. aeruginosa* rods are naturally resistant to a number of antimicrobials and relatively easily acquire other antibiotic resistance mechanisms. Resistance to antimicrobials results mostly from antibiotics-hydrolyzing enzymes synthesis, a loss of the OprD protein porins responsible for compound transport into bacterial cells, overexpression of a number of efflux pumps that pump out antimicrobials outside the cell or some other mechanisms [5].

*P. aeruginosa* strains’ pathogenicity depends on their ability to synthesize numerous enzymes (e.g., proteases, phospholipases), as well as cell- and biofilm-associated compounds (e.g., pili, alginate capsules) and extracellular toxins (e.g., exotoxins T, U and Y and exoenzyme S). Taken together, these play a potential role in infection development [6]. The mentioned virulence factors usually cooperate during *P. aeruginosa* colonization of the host organism at first, allowing for further infection and additionally increased pathogenicity [1].

The study was designed to determine the susceptibility profiles and genetic characteristics of 71 *P. aeruginosa* strains by assessing the distribution of 11 virulence-factor genes (*lasB*, *plC H*, *plC N*, *nan1*, *nan2*, *aprA*, *pilA*, *pilB*, *exoS*, *exoU* and *phzM*), as well as the composition of their genotypes. The mentioned genes were chosen based on the available literature and our preliminary studies [7,8] analyzing their presence among clinical strains derived exclusively from BSI cases. The genes were selected to include the most important types of *P. aeruginosa* virulence determinants, including those with enzymatic activity, those involved in extracellular toxin synthesis and factors contributing to bacterial colonization and biofilm synthesis. Therefore, the main aim of the study was to confirm the ability of different genotypes of *P. aeruginosa* isolates to cause development of bacteremia and to investigate the potential correlation between susceptibility profiles and genetic characteristics of *P. aeruginosa* strains in terms of their origin. 

## 2. Results

### 2.1. Antimicrobial Susceptibility of the Strains

**Figure 1:**
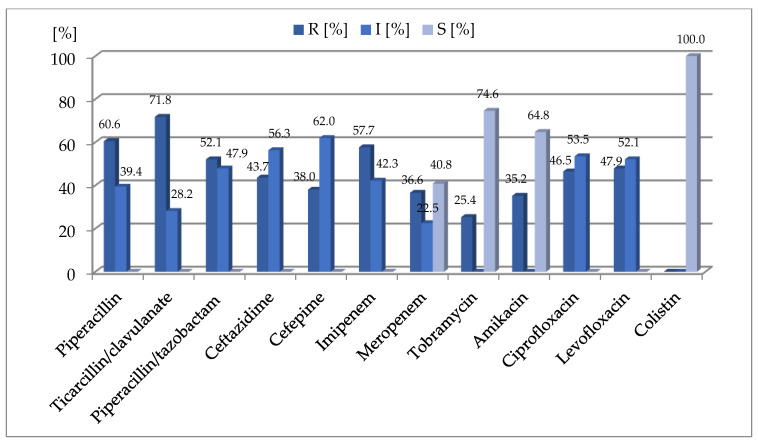
Susceptibility of the examined *P. aeruginosa* strains (*n* = 71). I: strains susceptible to antimicrobials at an increased exposure; R: strains resistant to antimicrobials; S: strains susceptible to antimicrobials at the standard doses.

All of the examined strains were susceptible to colistin. Most of the strains were resistant to ticarcillin/clavulanate (71.8%), piperacillin (60.6%), imipenem (57.7%) and piperacillin/tazobactam (52.1%). The detailed susceptibilities of the isolates included in the study are presented in Figure 1.

### 2.2. Prevalence of the Virulence-Factor Genes 

**Figure 2:**
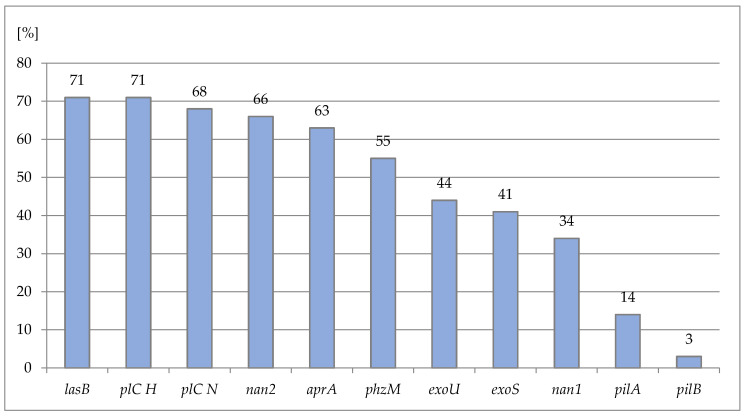
Prevalence of the studied genes in *P. aeruginosa* strains included in the study (*n* = 71). The numbers above the graph bars correspond to the numbers of strains positive for a particular gene.

An assessment of the virulence-factor genes’ frequency revealed a wide variety in gene distribution. The incidence of virulence genes amongst the examined strains was as follows: the *lasB* and *plC H* genes were noted in all of the tested strains, whereas the *pilA* and *pilB* genes were observed with the lowest frequency, in 19.7% and 4.2% of the studied isolates, respectively. The occurrences of the remaining virulence genes in the examined *P. aeruginosa* strains are shown in Figure 2.

Twenty-seven genotypes were observed (named I–XXVII); their prevalence and distribution amongst the examined strains are shown in Table 1. The most prevalent genotype, named IX and including all the genes detected except for the *exoU*, *pilA* and *pilB* genes, was observed in 13 (18.3%) isolates. Seventeen (24.9%) strains presented individual genotypes. The distribution of the genotypes detected amongst *P. aeruginosa* strains is presented in Table 1, their origin in Table S1, while the detailed specifications of the genotypes and strain susceptibilities are combined in Table S2.

### 2.3. Clinical Data Analysis 

A detailed clinical data analysis revealed that all the venous blood samples were collected from individuals suffering from fever. Specimens were collected at least twice from all the patients. All the positive blood cultures were initially checked microscopically for the presence of Gram-negative rods, and their existence was confirmed in each case. 

*P. aeruginosa* monoculture was obtained in 56 (78.9%) of the investigated bacteremia cases, while the presence of other microorganisms was confirmed for the remaining 15 (21.1%) blood samples (e.g., three cases accompanied by *Enterococcus faecium*, two by *Staphylococcus aureus*).

Before bacteremia development, forty-six (64.8%) patients presented symptoms of initial infections, and for all of these, positive *P. aeruginosa* cultures were obtained in the days preceding blood sample collection (bronchoalveolar lavage, wound swabs and urine samples). Primary infections were most likely to occur in the respiratory tract (29.6%), skin and soft tissues (7.0%), urinary tract (4.2%) or combined (23.9%) locations of initial infections. These cases were the most likely to develop subsequent bloodstream infections, but the route and development of BSIs as a secondary infection has not been determined. For the rest of the individuals, the primary site of infections remained unknown (either not investigated or possible primary BSIs).

Twenty-two (31.0%) patients included in the study at the time of blood sample collection were not subjected to antimicrobial treatment. The remaining patients were treated empirically, due to either primary infection or a probable bloodstream infection. Based on clinical experience, the applied drugs included those with anti-pseudomonal activity (mostly piperacillin/tazobactam, ceftazidime, imipenem or colistin) for 28 (39.4%) individuals and other antimicrobials for the remaining 21 (29.6%) patients. The patient survival rate over one month was confirmed for 41 (57.7%) patients included in the study.

### 2.4. Statistical Analysis 

A statistical analysis revealed a moderate positive correlation for the coexistence of the *aprA*/*nan2* pair of genes (*r_s_ =* 0.598298) and a weak positive correlation for a number of other gene pairs (the detailed results are presented in Table S3). In turn, the highest negative (although only moderate) correlation value was noted between *exoS* and *exoU* genes (*r_s_ =* −0.670076). The statistical calculations for the selected pairs of genes included into the study are presented in Table S3).

A statistically significant correlation was found for the frequency of *phzM* gene occurrence and patient survival over a month (Fisher’s exact test, *p* = 0.0278). Such relationships were not found for the remaining investigated virulence genes.

No statistically significant differences were found between the patients’ survival over one month and the probable origin of *P. aeruginosa* bacteremia or the applied empiric treatment involving antimicrobials with/without anti-*P. aeruginosa* activity. 

## 3. Discussion

The incidence of infection caused by clinical strains of bacteria in a specific location is determined by the presence and expression of particular virulence-factor genes. Individual isolates of the same species are generally characterized by a different pathogenic potential. Their level of virulence potential correlates with a different effect on the host organism. This also applies to *P. aeruginosa* strains. Most of their virulence factors are chromosomally encoded, and their gene sequence has been determined in the *P. aeruginosa* PAO1 strain genome [9]. However, *P. aeruginosa* genome plasticity has been previously confirmed in a number of studies [10,11,12,13]. In this study, the frequencies of the chosen 11 virulence-factor genes were examined, and the strains selected for this study show a relatively high percentage of virulence-gene presence.

The opportunistic pathogen discussed in this study is an etiological factor of many infections with different localizations, including BSI as one of the most threatening. BSIs due to *P. aeruginosa* strains are currently believed to be one of the most dangerous healthcare-associated infections [2,3,4]. Therefore, strains of this species isolated from bacteremia cases were used in this study to assess their susceptibility profiles as well as their pathogenic potential. 

Genetic features of *P. aeruginosa* have been widely investigated for the strains isolated from the respiratory tract [13,14,15,16,17,18,19], urinary tract [20,21,22], wounds [10,18,23,24,25,26] or many different origins simultaneously [27]. Meanwhile, relatively little is known about the association between genetic features and *P. aeruginosa* strains’ ability to cause BSIs. Thus, the association between the possibility of causing bloodstream infections and the presence of virulence genes is still unclear and requires further study. Meanwhile, as has been previously confirmed [28], even host stress in response to environmental signals may lead to expression of selected virulence determinants, which may cause lethal gut-derived sepsis. Thus, *P. aeruginosa* appears to be an example of colonizing pathogens which may cause development of a bloodstream infection as a response to surgical stress [28]. Moreover, *P. aeruginosa* is an example of bacteria that demonstrate the expression of many virulence factors, depending on the iron homeostasis of a host organism. Survival and further multiplication of these rods in a bloodstream depend on both the immune status of a host and the biological characteristics of the bacteria [29].

To the best of our knowledge, this is the first study that gives an insight into the susceptibility profiles in a relatively numerous collection of *P. aeruginosa* strains isolated exclusively from bacteremia cases, with respect to the genetic composition of the strains. 

The mortality of patients suffering from BSIs with *P. aeruginosa* might be diverse (even up to two-fold higher), depending on particular genetic features of strains, their susceptibility to carbapenems, for example, and bacterial persistence in clinical settings [30]. Higher mortality rates are generally found among patients with bacteremia caused by carbapenem-resistant *P. aeruginosa* strains, associated independently with extrinsic risk factors [31]. Although mortality indicators and BSI risk factors were investigated in the present study only in general, the percentage of carbapenem-resistant strains reached 57.7% and 36.6% for imipenem and meropenem, respectively. Therefore, this may have influenced the patients’ survival rate significantly. Interestingly, the corresponding values for carbapenem resistance amongst *P. aeruginosa* strains isolated from invasive infections in Europe, according to EARS-Net, were significantly lower at 16.5–19.3% between 2015 and 2019 (reaching up to 55.4% for selected countries) [3].

Apart from carbapenems, the percentages of *P. aeruginosa* antimicrobial-resistant isolates in the present study were generally higher than in the corresponding EARS-Net data. For example, average resistance levels observed for piperacillin/tazobactam (52.1% vs. over 12%), ceftazidime (43.7% vs. over 12%), fluoroquinolones (around 47% vs. 20%) and aminoglycosides (around 30% vs. over 12%) were also much higher, which is a very alarming situation.

The strains included in the research presented a relatively large variety of genotypes and corresponding antimicrobial susceptibility profiles. However, to the best of our knowledge, the only correlation between antibiotic resistance and a specific virulence gene was found between the *exoU* gene and the presence of the fluoroquinolones resistance mechanism [32]. In the current research, due to the presence of several dozen genotypes with a small number of strains in each genotype, it was not possible to determine a statistically significant relationship between the presence of the genotypes and the patterns of sensitivity to antibiotics.

In the available literature, numerous researchers have described the genetic features of *P. aeruginosa* with respect to different variables (e.g., strain origin from clinical and environmental sources, susceptibility to antimicrobials, clinical specimen type and patients’ hospitalization length or mortality) [7,30,33,34,35,36]. However, little information on the susceptibility patterns and especially the virulence-factor gene prevalence and genotypes distribution amongst the *P. aeruginosa* strains isolated exclusively from bacteremia can be found in the relevant literature. In the present study, the antimicrobial susceptibility profiles and 11 genes of the virulence factors or enzymes involved in their biosynthesis were evaluated. 

Interestingly, none of the strains among the examined isolates group carried all of the genes for the studied virulence factors. Additionally, as has been previously confirmed, *P. aeruginosa* virulence phenotypes correlate with virulence genotypes (e.g., for the type three secretion system, TTSS) and with resistance profiles but are rather a poor prognostic marker of mortality in bloodstream infections [37].

The most common *P. aeruginosa* virulence-factor genes detected in our study amongst all the analyzed strains were the *lasB* and *plC H* genes. It should be noted that the presence of the mentioned genes, as well as the positive correlation observed for the *nan2* and *aprA* genes’ coexistence, suggest that there might be a relationship between the carriage of these genes and the pathogenic potential of *P. aeruginosa* to cause the development of bacteremia cases. Meanwhile, the carriage of *pilA* and *pilB* genes was noted among only a small percentage of the strains with a bloodstream origin. In our opinion, this finding is very interesting, suggesting that the presence of particular genes is not crucial for the development of bacteremia following human colonization. There may be some other relationships between the carriage of the selected virulence genes and the ability of *P. aeruginosa* strains to colonize a particular individual, but this finding requires further study.

As was previously confirmed, there is an extensive conservation of virulence genes across the BSI-derived *P. aeruginosa* isolates and no very clear relation to patients’ mortality [38]. For example, in the cited study, the *exoU* gene was found in two isolates from patients who died rapidly and in one isolate from a patient that survived one year post BSI. Moreover, it was previously confirmed that the *exoU* genotype, which is associated with specific susceptibility profiles, is a relevant independent marker of early mortality in *P. aeruginosa* bacteremia [33]. Similar observations were not confirmed, either in the present research or for the *exoS* genotype. In fact, the pattern of immunological changes in septic patients suggests that quorum-sensing signal molecules are involved in the responses of the immune cells of the host and that signal molecules should be investigated as a cause of immune dysfunction in sepsis [39].

For *P. aeruginosa* strains isolated from invasive infections, the differences in virulence have not been well determined. It was previously observed that the virulence of ventilatory-associated pneumonia (VAP)-derived *P. aeruginosa* does not depend on biofilm formation, production of pyoverdine or the presence of some virulence genes, compared to *P. aeruginosa* isolated from non-invasive locations. However, this property of VAP-derived strains is not stable, and the strains show attenuated virulence compared to non-VAP counterparts in an in vivo model [40].

Whole-genome sequencing would be necessary to decipher the overall potential of antimicrobial resistance genes, multiple drug resistance and virulence-factor-encoding genes, as has recently been shown for one of the *P. aeruginosa* strains causing BSI [41]. The genome sequence of a recently identified and highly virulent *P. aeruginosa* PA45 strain was also provided by other researchers. Its 6.6-Mb genome, which contains 6822 genes, including an unparalleled number of virulence genes, might explain its aggressive phenotype [42]. Further studies would be necessary to analyze the characteristics of *P. aeruginosa* strains included in the present study, with their classification into sequence types.

The majority of pathogenic *P. aeruginosa* are capable of biofilm formation, mostly in the respiratory tract, as well as in medical and diagnostic equipment. As a result, they are more difficult to remove from the hospital environment. This virulence determinant is common in *P. aeruginosa* strains [35]. However, in the present study, the percentage of strains possessing the genes involved in colonization and biofilm synthesis (pilin-encoding genes) was the lowest. This may suggest that these particular genes are not crucial for causing bloodstream infections or that this particular group of strains follows an alternative route of BSI pathogenesis. Interestingly, it has previously been shown that a lack of motility independently increases the mortality of patients suffering from BSIs caused by *P. aeruginosa* [43]. 

Pilins genes, as determinants of bacterial biofilm formation, were observed at relatively low percentages (*pilA*—19.7% and *pilB*—4.2%) among the studied *P. aeruginosa* strains. These results are comparable with the results noted for *P. aeruginosa* strains derived from patients with a UTI [20]. In the mentioned study, 19.2% of the isolates carried the *pilA* gene, while none of the strains were positive for the *pilB* gene [20]. Another study by Sharifi et al. showed that the percentage of the *pilB* gene reached 8.3% in a group of *P. aeruginosa* isolates [44]. Only two of the strains studied in the present work carried both of the mentioned genes. These results indicate that for the evaluated strains group, the pathogenic potential and the major determinants involved in biofilm formation, observed at least on a molecular level, are not crucial for the development of bloodstream infections. Interestingly, the coexistence of the *pilA* and *pilB* genes was noted, suggesting that *P. aeruginosa* strains with this genotype are also capable of bacteremia development.

Almost 78% of the investigated isolates were positive for the *phzM* gene. This may suggest pyocyanin as one of the most prevalent virulence determinants of the strains isolated from bloodstream infections. Note that Fuse et al. showed that in a group of multidrug-resistant *P. aeruginosa* strains, the synthesis of pyocyanin decreased; however, this was not investigated in the present study [45]. Among the investigated genes, a statistically significant correlation was found only in the frequency of *phzM* gene occurrence and patients’ survival over a month. This is inconsistent with the results of the research conducted by Gupte et al. [43] in which pyocyanin synthesis was an independent factor promoting septic shock and an increased death rate in patients suffering from *P. aeruginosa* BSIs.

In the results of our study, a relatively high prevalence of the TTSS genes (*exoU*—62.0% and *exoS*—57.7%) was observed. The data from a recent meta-analysis on *P. aeruginosa* isolates with toxin potency indicated a relatively high prevalence of exotoxins in *P. aeruginosa* clinical isolates, but not exclusively in bacteremia cases [46]. In turn, the ability to synthesize toxins, especially in bacteremia cases, is of fundamental importance from a clinical point of view. However, this observation requires further study.

The results for *exoS* gene presence in our study are also concordant with the observations made by Khodayary et al. [47] and relatively close to those of Sharifi et al. [44], where the percentage of the *exoS* gene in a group of the examined multi-drug-resistant *P. aeruginosa* isolates (but not for the strains derived exclusively from BSIs) reached 59.0% and 44.0%, respectively. The study by Khodayary et al. also showed a high frequency of TTSS genes among *P. aeruginosa* strains, with the *exoU* gene noted among 41.0% of the isolates [47]. Interestingly, as previously indicated, the occurrence of a particular TTSS gene is associated with an increased resistance to antibiotics, at least amongst the strains derived from burn patients. Note that 62.0% of the isolates in this study carried the *exoU* gene. This observation is concordant with the results of previous research, although previous studies were not performed exclusively on bloodstream-derived isolates, e.g., a meta-analysis on the *P. aeruginosa exoU* gene with a value of.32 (CI 95%: 0.24–0.41) [46].

The presence of the *nan1* gene, noted in 47.9% of the studied strains, revealed the highest number of statistically important correlations, mostly positive. It may suggest that this particular gene only accompanies other genes’ presence, which is an interesting and unclear finding that also requires further research.

To summarize, great genetic diversity is observed amongst *P. aeruginosa* strains isolated from bloodstream infections. This variability is probably based on the presence of a particular plasmid or chromosomal gene set. The cause of this diversity might be the genetic material reorganization, e.g., conserving the genes crucial for colonization and development of infection in the hospital environment (e.g., the *lasB* or *plC H* genes) and losing the currently less important virulence factors at the particular infection stage (e.g., pilin-encoding genes).

The frequency of genes encoding virulence factors and their synthesis affects the pathogenesis of *P. aeruginosa* infection and may lead to a more severe course of bloodstream infection. Mechanisms influencing the host’s immune system response play a significant role during bacteremia; thus, among the tested isolates, a high percentage of genes encoding factors contributing to immunomodulation were noted, i.e., *lasB*, *plC H*, *plC N*, *aprA*, *exoS* and, *exoU*.

However, as was shown for the reference strain, the mentioned properties can also be achieved through the particular expression of relevant genes [48]. Substantial changes in the transcriptome of the strains are observed [49], including metabolic changes and the alteration of virulence, for example, during bloodstream infections [50]. These results suggest that, as part of its survival strategy and in response to changes in the patients’ blood, *P. aeruginosa* enhances the expression of certain virulence genes and reduces the expression of others [49,50].

This study is believed to be one of the first and largest evaluations of the carriage of virulence-factor genes in a group of *P. aeruginosa* strains isolated exclusively from bacteremia cases. It reports on the diverse susceptibility of *P. aeruginosa* strains, as well as their virulence potential on a genetic level. Further studies are required to understand the mechanism of *P. aeruginosa* virulence genes’ carriage and their direct involvement in the BSIs pathogenicity process, as previously suggested [51].

The limitations of the study were as follows: a relatively small number of strains were included in the study, which also limited the statistical evaluation of the results, and a subjective selection of the virulence genes was made, based on the results of our own previous investigations as well as the relevant scientific literature in the field of *P. aeruginosa* genetic studies. 

The results of the evaluation of a particular gene’s presence among the bloodstream-derived isolates could be used to develop a new time-saving bacteremia diagnostic approach, e.g., a PCR test based on particular gene targeting, preceding or without the necessity for the strain culture. This would also help to improve the treatment policies against *P. aeruginosa* bacteremia amongst hospitalized patients, e.g., via immediate application of a semi-targeted antimicrobial treatment with a mode of action involving protein synthesis inhibition. In effect, it would provide the necessary tools for the rapid diagnosis of infections caused by *P. aeruginosa* virulent strains and allow the patient’s condition to be monitored and improved. Finally, it could help to establish better strategies for *P. aeruginosa* bloodstream infection treatment and probably to decrease the mortality of patients suffering from *P. aeruginosa* bacteremia.

Further studies are needed to decipher the virulent genetic potential or epigenetic features of the isolates with this origin, with respect to the carriage of particular resistance mechanisms and their pathogenic properties.

## 4. Materials and Methods

### 4.1. Origin of the Strains, Their Selection Criteria and Clinical Data Analysis

In total, 71 clinical isolates of *P. aeruginosa* strains were included in the study. The strains were initially identified based on typical growth on a selective medium (Cetrimide agar, *bio*Mérieux, Marcy-l’Étoile, France) and biochemical reaction results (oxidase, catalase). The final identification was made using a MALDI-TOF MS method performed on a MALDI Biotyper device (Bruker, Mannheim, Germany). All the strains were isolated and collected in the Clinical Microbiology Laboratory of Dr. Antoni Jurasz University Hospital No. 1 in Bydgoszcz, Poland. The majority of the strains were derived from patients in the Anesthesiology and Intensive Care Unit (47.9%). All the isolates included in the study were derived from blood samples of patients with microbiologically and clinically confirmed bacteremia, with each strain from a different patient. The blood pre-cultures were obtained in an automatic system for blood and body fluids monitoring (BACTEC™ FX, Becton Dickinson, Heidelberg, Germany).

Clinical data were also analyzed in detail, including empiric antimicrobial treatment at the time of blood sample collection, an analysis of the primary infection site, the presence of other microorganisms in the samples and patients’ survival rate over one month.

### 4.2. Antimicrobial Susceptibility Testing

During the antimicrobial susceptibility testing (AST) step, the following methods were used: the disc diffusion method on Mueller–Hinton agar (Becton Dickinson, Heidelberg, Germany) for ticarcillin/clavulanate, microdilution colistin MIC evaluation tests (SensiTest Colistin, Liofilchem, Roseto degli Abruzzi, Italy) and NMIC-402 panels of a Phoenix^TM^ M50 device (Becton Dickinson, Germany) for the remaining antimicrobials. Although the strains were derived between 2015 and 2019, the results of the AST were interpreted according to the current European Committee on Antimicrobial Susceptibility Testing Recommendations (EUCAST, breakpoint tables for bacteria, clinical breakpoints—bacteria v 12.0, 2022) to unify the strains’ susceptibility categories. *P. aeruginosa* obtained from the American Type Culture Collection (ATCC 27853), as well as *Escherichia coli* strains ATCC 25922 and 35218, served as AST quality controls.

### 4.3. Bacterial DNA Isolation

A Genomic Mini kit (A&A Biotechnology, Gdynia, Poland) was used for the isolation of DNA samples, performed according to the manufacturer’s protocol. All the DNA samples were stored at 4 °C before further use for the purposes of the study.

### 4.4. Virulence-Factor Genes Detection

The prevalence of 11 virulence-factor genes was determined by PCR, in a separate reaction for each gene. The genes were amplified with primers selected on the basis of the published *P. aeruginosa* PAO1 strain genome sequence and the *P. aeruginosa* ATCC 27853 isolate, and the amplification procedure was carried out as previously described [13,15,52]. The reactions were performed in 0.2 mL test tubes (Eppendorf, Hamburg, Germany), with a final volume of 20 μL. Briefly, *Taq* polymerase was used with total activity of 1 U per reaction in a 1 × concentrated polymerase buffer with MgCl_2_ at a final concentration of 1.5 mM (Go *Taq* G2 Polymerase, Promega, Walldorf, Germany or FirePol DNA Polymerase, Solis BioDyne, Tartu, Estonia) and deoxynucleotide triphosphates (dNTPs) set at a final concentration of 200 μM (Promega, Germany, Solis BioDyne, Estonia). The primers were used in a final amount of 12.5 pmol per reaction (Integrated DNA Technologies, Coralville, IA, USA, Sigma, Darmstadt, Germany or Genomed, Poland). The primers’ sequences and the PCR annealing temperatures for each gene amplification are presented in Table 2. The DNA isolated from the *P. aeruginosa* PAO1 strain (kindly provided by the National Medicines Institute in Warsaw, Poland) and the *P. aeruginosa* ATCC 27853 isolate served as a PCR positive control. In the amplification procedure, a GeneAmp^®^ PCR System 2700 thermal cycler (Applied Biosystems, Foster City, CA, USA) was applied. The presence of amplicons for the particular genes was evaluated visually with the application of gel electrophoresis (1.5% agarose, Bio-Rad, Feldkirchen, Germany), based on the product size and the control-strain DNA amplification (Figures S1–S11). For the selected, arbitrarily chosen samples, PCR duplicates were performed to confirm the repeatability of the results, giving consistent results in each case. 

### 4.5. Statistical Methods

Statistical analyses were performed using the StatSoft Inc., (Tulsa, OK, USA,) STATISTICA 13.3 (2017) (data analysis software system) program, using Spearman’s rank-order correlation test to investigate the correlation for particular genes’ coexistence (*p* ≤ 0.05). 

A chi-squared test was also used to establish statistically significant relationships between the following features (α = 0.05): (i) patients’ survival over a month and the probable initial site of *P. aeruginosa* infection/origin of bacteremia, (ii) the frequency of a particular gene occurrence and patients’ survival over one month (Fisher’s exact test) and (iii) patients’ survival over a month and the initially applied antibiotic with/without anti-*P. aeruginosa* activity. Fisher’s exact test was used each time if the assumptions of the chi-squared test were not met.

Note that due to the presence of several dozen genotypes with a small number of strains in each genotype, it was not possible to determine the relationship between the presence of a given set of genes and the sensitivity to antibiotics. 

## 5. Conclusions

A very high genetic diversity was noted amongst the *P. aeruginosa* strains isolated from bacteremia cases; however, all the strains isolated from bloodstream infections possessed *lasB* and *plC H* genes. Therefore, both of these genes might be used as molecular diagnostic biomarkers for the detection of *P. aeruginosa* strains causing bacteremia. 

No statistically significant differences were found between the patients’ survival over one month and the possible origin of *P. aeruginosa* bacteremia or the applied empiric treatment involving antimicrobials with anti-pseudomonal activity.

It is likely that a diverse virulence potential of *P. aeruginosa* strains causing bloodstream infections may result from a loss of particular genes that are not crucial for the development of bacteremia. For example, genes encoding *P. aeruginosa* pilins were found at the lowest frequency for this particular strain group, suggesting that biofilm- and/or colonization-associated properties involve other virulence factors or follow other pathomechanisms in the development of BSIs. 

## Figures and Tables

**Table 1:** Characteristics and distribution of the genotypes detected amongst the examined *P. aeruginosa* strains (*n* = 71).

*lasB*	*plC H*	*plC N*	*nan2*	*aprA*	*phzM*	*exoU*	*exoS*	*nan1*	*pilA*	*pilB*	Genotype Number	Number of Strains (*n* = 71)	Percentage of Strains (%)
+	+	+	+	+	+	+	+	+	+	-	I	2	2.8
+	+	+	+	+	+	+	+	+	-	-	II	2	2.8
+	+	+	+	+	+	+	+	-	-	-	III	4	5.6
+	+	+	+	+	+	+	-	+	+	-	IV	5	7.0
+	+	+	+	+	+	+	-	+	-	-	V	5	7.0
+	+	+	+	+	+	+	-	-	+	+	VI	1	1.4
+	+	+	+	+	+	+	-	-	-	-	VII	10	14.1
+	+	+	+	+	+	-	+	+	+	-	VIII	1	1.4
+	+	+	+	+	+	-	+	+	-	-	IX	13	18.3
+	+	+	+	+	+	-	+	-	-	-	X	6	8.5
+	+	+	+	+	-	+	+	-	-	-	XI	1	1.4
+	+	+	+	+	-	+	-	-	-	+	XII	1	1.4
+	+	+	+	+	-	+	-	-	-	-	XIII	4	5.6
+	+	+	+	+	-	-	+	+	-	-	XIV	3	4.2
+	+	+	+	+	-	-	+	-	+	-	XV	1	1.4
+	+	+	+	+	-	-	+	-	-	-	XVI	1	1.4
+	+	+	+	-	+	+	+	+	+	-	XVII	1	1.4
+	+	+	+	-	+	+	+	+	-	-	XVIII	1	1.4
+	+	+	+	-	+	-	+	-	+	+	XIX	1	1.4
+	+	+	+	-	-	+	+	-	-	-	XX	1	1.4
+	+	+	-	+	+	+	-	-	-	-	XXI	1	1.4
+	+	+	-	-	-	+	+	-	-	-	XXII	1	1.4
+	+	+	-	-	-	+	-	-	-	-	XXIII	1	1.4
+	+	+	-	-	-	-	+	-	-	-	XXIV	1	1.4
+	+	-	+	+	+	+	+	+	+	-	XXV	1	1.4
+	+	-	+	+	+	+	-	-	+	-	XXVI	1	1.4
+	+	-	-	-	-	+	-	-	-	-	XXVII	1	1.4

(+): Presence of a particular gene (shaded boxes); (-): absence of a particular gene.

**Table 2:** Specification of PCR primers and parameters applied in the present study.

Virulence Factor Detected	Gene/PCR Primer Name	Manufacturer	Primer Sequence 5′→3′	Tm (°C)	Annealing Temperature (°C)	Amplicon Size (bp)
Exotoxin U	*exoU F*	Sigma	CCGTTGTGGTGCCGTTGAAG	55.9	64	134
	*exoU R*		CCAGATGTTCACCGACTCGC	55.9		
Exoenzyme S	*exoS F*	Integrated DNA Technologies	CTTGAAGGGACTCGACAAGG	55.2	53	504
	*exoS R*		TTCAGGTCCGCGTAGTGAAT	56.2		
Phospholipase C (non-hemolytic)	*plC N F*		GTTATCGCAACCAGCCCTAC	55.9	53	466
	*plC N R*		AGGTCGAACACCTGGAACAC	57.2		
Phospholipase C (hemolytic)	*plC H F*		GAAGCCATGGGCTACTTCAA	55.1	52	307
	*plC H R*		AGAGTGACGAGGAGCGGTAG	58.2		
Elastase B	*lasB F*	Genomed	GGAATGAACGAAGCGTTCTC	51.8	50	300
	*lasB R*		GGTCCAGTAGTAGCGGTTGG	55.9		
Phenazine methyltransferase	*phzM F*		ATGGAGAGCGGGATCGACAG	55.9	54	875
	*phzM R*		ATGCGGGTTTCCATCGGCAG	55.9		
Pilin A	*pilA F*		ACAGCATCCAACTGAGCG	50.3	59	1675
	*pilA R*		TTGACTTCCTCCAGGCTG	50.3		
Pilin B	*pilB F*		TCGAACTGATGATCGTGG	48.0	54	408
	*pilB R*		CTTTCGGAGTGAACATCG	48.0		
Neuraminidase 1	*nan1 F*		AGGATGAATACTTATTTTGAT	42.6	47	1316
	*nan1 R*		TCACTAAATCCATCTCTGACCCGATA	56.4		
Neuraminidase 2	*nan2 F*		GTTTTGCTGATGCTGGTTCA	51.1	50	1161
	*nan2 R*		TGTCCAGCAATTCTCTTGC	49.7		
Alkaline protease	*aprA F*		TGTCCAGCAATTCTCTTGC	48.9	50	1017
	*aprA R*		CGTTTTCCACGGTGACC	49.5		

## Data Availability

The data presented in this study are available on request from the corresponding author.

## Supplementary Materials

The following are available online at https://www.mdpi.com/article/10.3390/ijms23169208/s1.
