# Supplementary material for: Decoding Genetic Features and Antimicrobial Susceptibility of Pseudomonas aeruginosa Strains Isolated from Bloodstream Infections
Source: Int J Mol Sci. 2022 Aug 16;23(16):9208. doi: 10.3390/ijms23169208 (PMC9409454; doi:10.3390/ijms23169208)
Supplement: Supplementary file 1 [file ijms-23-09208-s001.zip › ijms-1845894-SI.pdf]

Decoding genetic features and antimicrobial susceptibility of *Pseudomonas aeruginosa* strains isolated from bloodstream infections

Tomasz Bogiel*, Dagmara Depka, Mateusz Rzepka and Agnieszka Mikucka

Microbiology Department, Ludwik Rydygier Collegium Medicum in Bydgoszcz, Nicolaus Copernicus University in Toruń, 85-094 Bydgoszcz, Poland
* Correspondence: e-mail: t.bogiel@cm.umk.pl, telephone number: +48 52 585 44 80

Table S1. The origin of the *P. aeruginosa* strains included into the study (*n* = 71)

**Department/Clinic**	**Number of strains (*n* = 71)**	**Percentage of strains (%)**
Department of Anaesthesiology and Intensive Care	34	47.9
Department of Cardiology and Internal Medicine	14	19.7
Clinical Unit of Paediatric Anaesthesiology and Intensive Therapy	6	8.5
Department of Paediatrics, Haematology and Oncology with Bone Marrow Transplant Clinic	5	7.0
Department of Liver and General Surgery	3	4.2
Department of Geriatrics	2	2.8
Department of Nephrology, Hypertension and Internal Medicine and Dialysis Station	2	2.8
Department of Cardiac Surgery and Intensive Care	1	1.4
Department of Transplantation and General Surgery	1	1.4
Department of General, Oncologic and Paediatric Urology	1	1.4
Department of Dermatology, Sexually Transmitted Diseases and Immunodermatology	1	1.4
Department of Neurology	1	1.4

Table S2. The detailed distribution of genotypes with respect to susceptibility profiles of the *P. aeruginosa* strains included into the study (*n* = 71)

Strain No./characteristic	*lasB*	*plC H*	*plC N*	*nan2*	*aprA*	*phzM*	*exoU*	*exoS*	*nan1*	*pilA*	*pilB*	Genotype number	Number of strains (*n* = 71)	Percentage of strains (%)	Piperacillin	Ticarcillin/clavulanate	Piperacillin/tazobactam	Ceftazidime	Cefepime	Imipenem	Meropenem	Tobramycin	Amikacin	Ciprofloxacin	Levofloxacin	Colistin
P428	+	+	+	+	+	+	+	+	+	+	-	I	2	2.8	I	I	I	I	I	R	I	S	S	I	I	S
P459	+	+	+	+	+	+	+	+	+	+	-				R	R	I	I	I	I	S	S	S	R	R	S
P242	+	+	+	+	+	+	+	+	+	-	-	II	2	2.8	R	R	R	R	R	R	R	R	R	R	R	S
P306	+	+	+	+	+	+	+	+	+	-	-				R	R	R	R	R	I	S	S	S	I	I	S
P301	+	+	+	+	+	+	+	+	-	-	-	III	4	5.6	R	R	R	R	R	R	R	S	S	I	I	S
P453	+	+	+	+	+	+	+	+	-	-	-				R	R	R	R	R	I	S	S	R	R	R	S
P569	+	+	+	+	+	+	+	+	-	-	-				I	I	I	I	I	I	S	S	S	I	I	S
P575	+	+	+	+	+	+	+	+	-	-	-				I	I	I	I	I	I	S	S	S	I	I	S
P247	+	+	+	+	+	+	+	-	+	+	-	IV	5	7.0	I	I	I	I	I	R	R	S	S	R	R	S
P317	+	+	+	+	+	+	+	-	+	+	-				I	I	I	I	I	I	S	S	S	I	I	S
P318	+	+	+	+	+	+	+	-	+	+	-				R	R	R	R	R	R	R	S	S	R	R	S
P450	+	+	+	+	+	+	+	-	+	+	-				I	I	I	I	I	R	S	S	S	I	R	S
P567	+	+	+	+	+	+	+	-	+	+	-				I	R	I	I	I	R	R	S	S	I	I	S
P315	+	+	+	+	+	+	+	-	+	-	-	V	5	7.0	R	R	R	R	R	R	R	S	S	I	I	S
P346	+	+	+	+	+	+	+	-	+	-	-				I	R	I	I	I	R	I	S	S	I	I	S
P449	+	+	+	+	+	+	+	-	+	-	-				R	R	R	R	I	R	I	S	S	I	I	S
P455	+	+	+	+	+	+	+	-	+	-	-				R	R	R	R	I	I	S	S	S	I	I	S
P547	+	+	+	+	+	+	+	-	+	-	-				R	I	R	I	I	R	I	S	S	I	I	S
P395	+	+	+	+	+	+	+	-	-	+	+	VI	1	1.4	I	I	I	I	I	I	S	S	S	I	I	S
P175	+	+	+	+	+	+	+	-	-	-	-	VII	10	14.1	R	R	R	R	R	R	R	R	R	R	R	S
P245	+	+	+	+	+	+	+	-	-	-	-				R	R	R	R	R	R	R	R	R	R	R	S
P246	+	+	+	+	+	+	+	-	-	-	-				R	R	I	R	I	R	R	R	R	I	I	S
P248	+	+	+	+	+	+	+	-	-	-	-				R	R	R	R	I	R	R	R	R	R	R	S
P249	+	+	+	+	+	+	+	-	-	-	-				R	R	R	R	R	R	R	S	S	I	I	S
P251	+	+	+	+	+	+	+	-	-	-	-				R	R	R	R	I	R	R	R	R	R	R	S
P300	+	+	+	+	+	+	+	-	-	-	-				R	R	R	R	R	R	R	R	R	R	R	S
P308	+	+	+	+	+	+	+	-	-	-	-				R	R	R	R	R	R	R	R	R	R	R	S
P568	+	+	+	+	+	+	+	-	-	-	-				R	R	R	R	R	R	R	R	R	R	R	S
P576	+	+	+	+	+	+	+	-	-	-	-				R	R	R	R	I	R	R	R	S	R	R	S
P447	+	+	+	+	+	+	-	+	+	+	-	VIII	1	1.4	R	R	R	R	R	I	S	S	S	I	I	S
P170	+	+	+	+	+	+	-	+	+	-	-	IX	13	18.3	R	R	I	I	I	I	S	S	R	R	R	S
P171	+	+	+	+	+	+	-	+	+	-	-				R	R	R	R	I	R	I	S	R	R	R	S
P172	+	+	+	+	+	+	-	+	+	-	-				R	R	I	I	I	I	S	S	R	R	R	S
P174	+	+	+	+	+	+	-	+	+	-	-				I	I	I	I	I	I	S	S	R	R	R	S
P241	+	+	+	+	+	+	-	+	+	-	-				R	R	R	R	R	R	I	R	R	R	R	S
P244	+	+	+	+	+	+	-	+	+	-	-				R	R	R	R	R	R	I	R	R	R	R	S
P302	+	+	+	+	+	+	-	+	+	-	-				R	R	R	R	R	R	R	S	S	I	I	S
P303	+	+	+	+	+	+	-	+	+	-	-				R	R	R	R	R	I	S	S	S	I	I	S
P304	+	+	+	+	+	+	-	+	+	-	-				I	R	I	I	I	R	I	S	R	R	R	S
P305	+	+	+	+	+	+	-	+	+	-	-				R	R	R	R	R	R	R	S	S	I	I	S
P310	+	+	+	+	+	+	-	+	+	-	-				R	R	R	R	R	R	R	S	S	R	R	S

P311	+	+	+	+	+	+	-	+	+	-	-				R	R	R	I	I	I	S	S	S	I	I	S
P458	+	+	+	+	+	+	-	+	+	-	-				R	R	I	I	I	I	S	S	R	R	R	S
P309	+	+	+	+	+	+	-	+	-	-	-	X	6	8.5	R	R	I	I	I	R	I	S	S	R	R	S
P313	+	+	+	+	+	+	-	+	-	-	-				R	R	R	R	R	R	R	S	S	R	R	S
P314	+	+	+	+	+	+	-	+	-	-	-				I	I	I	I	I	I	S	S	S	I	I	S
P316	+	+	+	+	+	+	-	+	-	-	-				I	I	I	I	I	I	S	S	S	I	I	S
P319	+	+	+	+	+	+	-	+	-	-	-				I	I	I	I	I	I	S	S	S	I	I	S
P454	+	+	+	+	+	+	-	+	-	-	-				I	I	I	I	I	I	S	S	S	I	I	S
P574	+	+	+	+	+	-	+	+	-	-	-	XI	1	1.4	I	R	I	I	I	R	I	S	S	I	I	S
P571	+	+	+	+	+	-	+	-	-	-	+	XII	1	1.4	I	R	I	I	I	I	S	S	S	I	I	S
P252	+	+	+	+	+	-	+	-	-	-	-	XIII	4	5.6	I	I	I	I	I	R	I	S	S	I	I	S
P451	+	+	+	+	+	-	+	-	-	-	-				R	R	R	R	R	R	R	R	R	R	R	S
P456	+	+	+	+	+	-	+	-	-	-	-				R	R	R	I	R	R	I	R	R	R	R	S
P457	+	+	+	+	+	-	+	-	-	-	-				R	R	R	R	R	R	R	R	R	R	R	S
P307	+	+	+	+	+	-	-	+	+	-	-	XIV	3	4.2	I	I	I	I	I	R	I	S	S	R	R	S
P312	+	+	+	+	+	-	-	+	+	-	-				I	R	I	I	I	I	S	S	S	I	I	S
P452	+	+	+	+	+	-	-	+	+	-	-				I	I	I	I	I	I	S	S	S	I	I	S
P04	+	+	+	+	+	-	-	+	-	+	-	XV	1	1.4	I	I	I	I	I	R	I	S	S	I	I	S
P448	+	+	+	+	+	-	-	+	-	-	-	XVI	1	1.4	R	I	R	I	I	I	I	R	R	R	R	S
P570	+	+	+	+	-	+	+	+	+	+	-	XVII	1	1.4	I	R	I	I	I	I	S	S	S	I	I	S
P243	+	+	+	+	-	+	+	+	+	-	-	XVIII	1	1.4	R	R	R	R	I	R	R	R	R	R	R	S
P173	+	+	+	+	-	+	-	+	-	+	+	XIX	1	1.4	R	R	R	I	R	R	R	S	R	R	R	S
P369	+	+	+	+	-	-	+	+	-	-	-	XX	1	1.4	I	I	I	I	I	I	S	S	S	I	I	S
P20	+	+	+	-	+	+	+	-	-	-	-	XXI	1	1.4	R	R	R	I	R	R	I	S	S	R	R	S
P502	+	+	+	-	-	-	+	+	-	-	-	XXII	1	1.4	I	I	I	I	I	I	S	S	S	I	I	S
P483	+	+	+	-	-	-	+	-	-	-	-	XXIII	1	1.4	I	R	I	I	I	I	S	S	S	I	I	S
P164	+	+	+	-	-	-	-	+	-	-	-	XXIV	1	1.4	I	R	I	I	I	I	S	S	S	I	I	S
P320	+	+	-	+	+	+	+	+	+	+	-	XXV	1	1.4	I	R	I	I	I	I	S	S	S	I	I	S
P563	+	+	-	+	+	+	+	-	-	+	-	XXVI	1	1.4	R	R	R	R	R	R	R	S	S	I	I	S
P119	+	+	-	-	-	-	+	-	-	-	-	XXVII	1	1.4	R	R	R	I	R	I	R	R	R	R	R	S

(+) – presence of a particular gene; shaded boxes, (-) – absence of a particular gene, I – strains susceptible to antimicrobials at increased exposure, R – strains resistant to antimicrobials, S – strains susceptible to antimicrobials at the standard doses

Table S3: The distribution and statistically significant correlation revealed for the selected pairs of genes included into the study

Gene pairs	No. (%) of the isolates with a particular gene presence (*n* = 71)				r_s
	+/+	+/–	–/+	–/–	
aprA/*nan2*	62 (87.3)	4 (5.6)	1 (1.4)	4 (5.6)	0.598298
phzM/*nan2*	54 (76.1)	12 (16.9)	1 (1.4)	4 (5.6)	0.378558
phzM/*aprA*	52 (73.2)	3 (4.2)	11 (15.5)	5 (7.0)	0.340855
nan1/*phzM*	31 (43.7)	3 (4.2)	24 (33.8)	13 (18.3)	0.314591
nan1/*nan2*	34 (47.9)	0 (0.0)	32 (45.1)	5 (7.0)	0.263847
nan1/*exoS*	24 (33.8)	10 (14.1)	17 (23.9)	20 (28.2)	0.249212
pilA/*pilB*	2 (2.8)	12 (16.9)	1 (1.4)	56 (78.9)	0.247847
pilA/*nan1*	10 (14.1)	4 (5.6)	24 (33.8)	33 (46.5)	0.233547
nan1/*exoU*	17 (23.9)	17 (23.9)	27 (38.0)	10 (14.1)	-0.236401
pilA/*plC N*	12 (16.9)	2 (2.8)	56 (78.9)	1 (1.4)	-0.247847
exoS/*exoU*	14 (19.7)	27 (38.0)	30 (42.3)	0 (0.0)	-0.670076

(+) – presence of a particular gene; (-) – absence of a particular gene; r_s – Spearman's rank correlation coefficient (< 0.2 – none or statistically irrelevant correlation (data not show), 0.2 – 0.4 a weak correlation, 0.4 – 0.7 – a moderate correlation, 0.7 – 0.9 a strong correlation, > 0.9 – a very strong correlation)

Figures S1-S11. Pictures of the electrophoretic gels showing the amplicons of PCR for the corresponding genes detected in the present study, with their decreasing frequency; (M - DNA size marker of 100-1.000 or 100-3.000 bp; numbers – names assigned to a particular strain; (+) or K(+) – positive PCR control; (-) or K(-) – negative PCR control

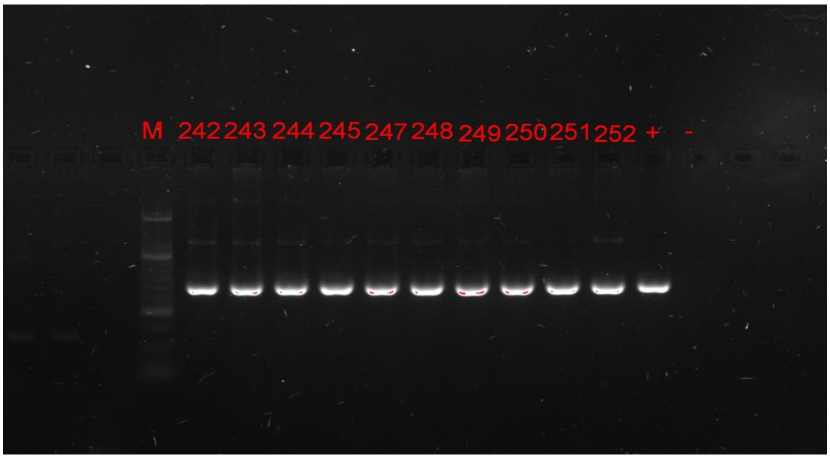

Figure S1 – the *lasB* gene

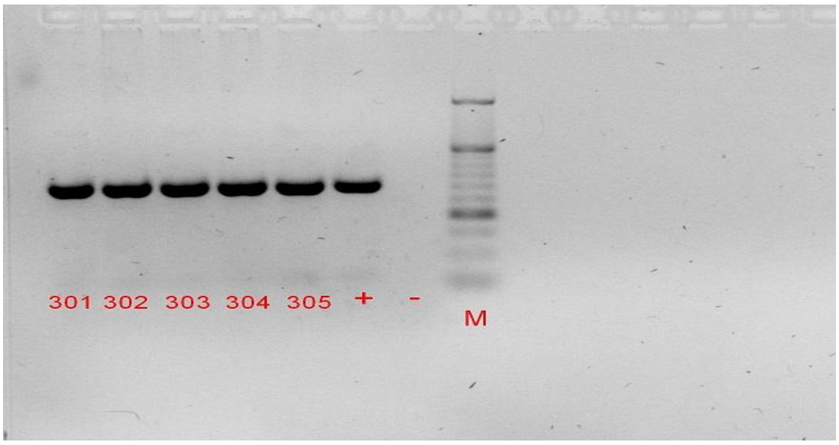

Figure S2 – the *plC H* gene

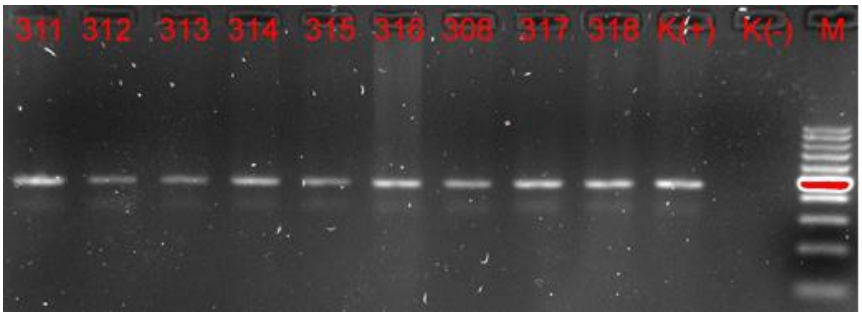

Figure S3 – the *plC N* gene

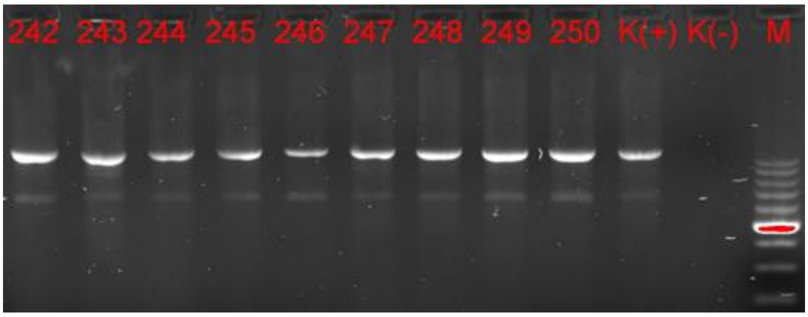

Figure S4 – the *nan2*gene

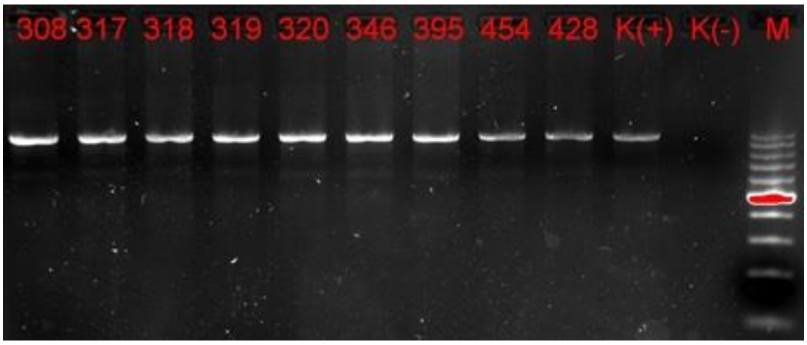

Figure S5 – the *aprA* gene

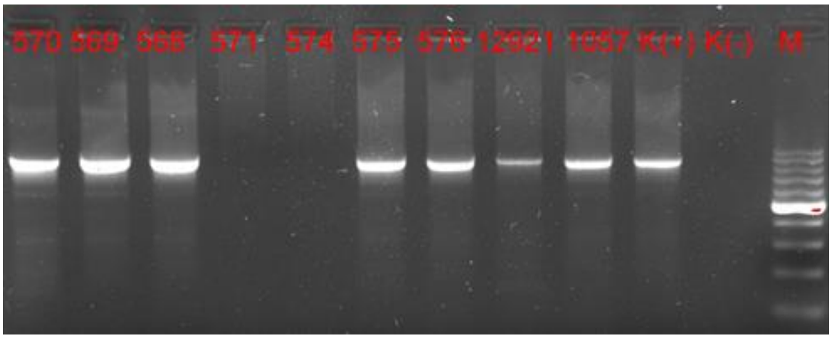

Figure S6 – the *phzM* gene

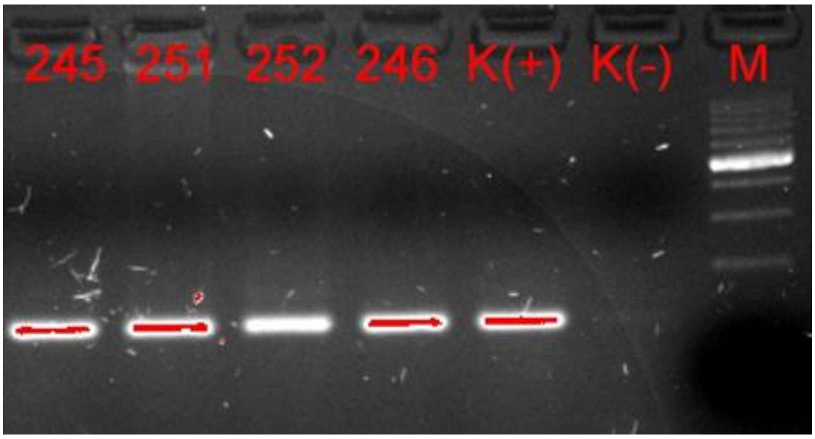

Figure S7 – the *exoU* gene

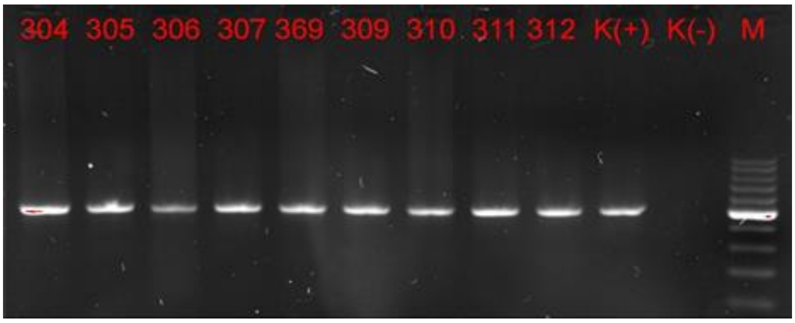

Figure S8 – the *exoS* gene

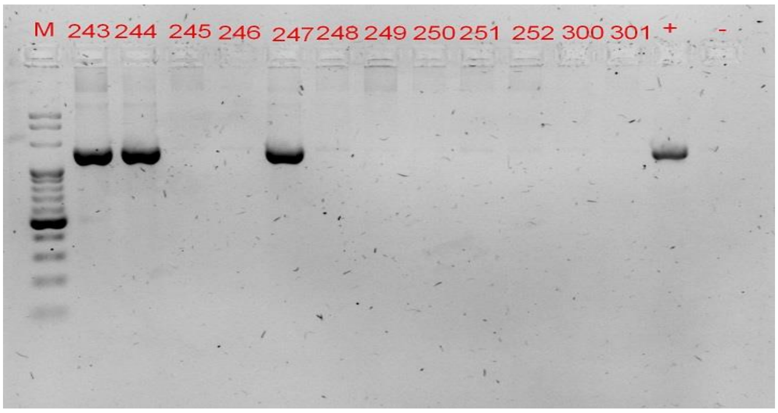

Figure S9 – the *nan1* gene

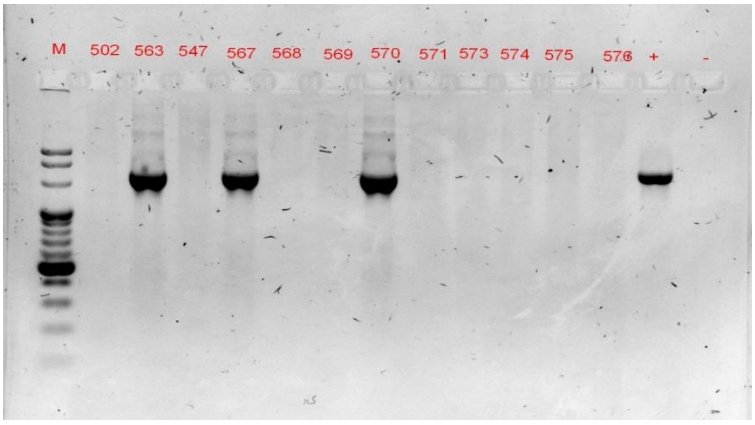

Figure S10 – the *pilA* gene

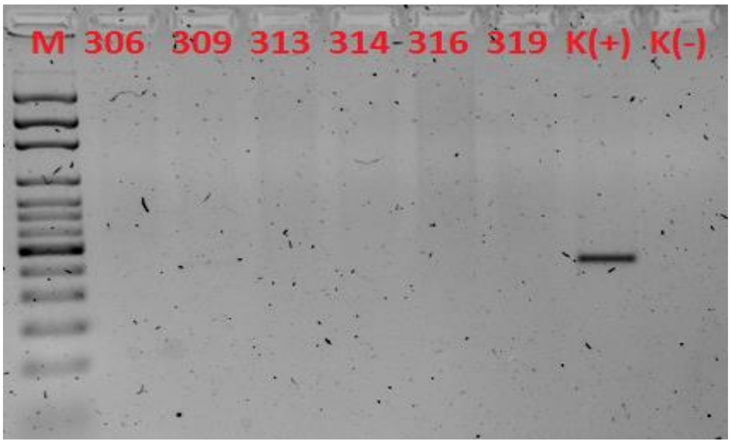

Figure S11 – the *pilB* gene
